# Morphological Plant Modeling: Unleashing Geometric and Topological Potential within the Plant Sciences

**DOI:** 10.3389/fpls.2017.00900

**Published:** 2017-06-09

**Authors:** Alexander Bucksch, Acheampong Atta-Boateng, Akomian F. Azihou, Dorjsuren Battogtokh, Aly Baumgartner, Brad M. Binder, Siobhan A. Braybrook, Cynthia Chang, Viktoirya Coneva, Thomas J. DeWitt, Alexander G. Fletcher, Malia A. Gehan, Diego Hernan Diaz-Martinez, Lilan Hong, Anjali S. Iyer-Pascuzzi, Laura L. Klein, Samuel Leiboff, Mao Li, Jonathan P. Lynch, Alexis Maizel, Julin N. Maloof, R. J. Cody Markelz, Ciera C. Martinez, Laura A. Miller, Washington Mio, Wojtek Palubicki, Hendrik Poorter, Christophe Pradal, Charles A. Price, Eetu Puttonen, John B. Reese, Rubén Rellán-Álvarez, Edgar P. Spalding, Erin E. Sparks, Christopher N. Topp, Joseph H. Williams, Daniel H. Chitwood

**Affiliations:** ^1^Department of Plant Biology, University of Georgia, AthensGA, United States; ^2^Warnell School of Forestry and Natural Resources, University of Georgia, AthensGA, United States; ^3^Institute of Bioinformatics, University of Georgia, AthensGA, United States; ^4^School of Forestry and Environmental Studies, Yale University, New HavenCT, United States; ^5^Laboratory of Applied Ecology, Faculty of Agronomic Sciences, University of Abomey-CalaviCotonou, Benin; ^6^Department of Biological Sciences, Virginia Polytechnic Institute and State University, BlacksburgVA, United States; ^7^Department of Geosciences, Baylor University, WacoTX, United States; ^8^Department of Biochemistry and Cellular and Molecular Biology, University of Tennessee, Knoxville, KnoxvilleTN, United States; ^9^The Sainsbury Laboratory, University of CambridgeCambridge, United Kingdom; ^10^Division of Biology, University of Washington, BothellWA, United States; ^11^Donald Danforth Plant Science Center, St. LouisMO, United States; ^12^Department of Wildlife and Fisheries Sciences–Department of Plant Pathology and Microbiology, Texas A&M University, College StationTX, United States; ^13^School of Mathematics and Statistics and Bateson Centre, University of SheffieldSheffield, United Kingdom; ^14^Department of Mathematics, Florida State University, TallahasseeFL, United States; ^15^Weill Institute for Cell and Molecular Biology and Section of Plant Biology, School of Integrative Plant Sciences, Cornell University, IthacaNY, United States; ^16^Department of Botany and Plant Pathology, Purdue University, West LafayetteIN, United States; ^17^Department of Biology, Saint Louis University, St. LouisMO, United States; ^18^School of Integrative Plant Science, Cornell University, IthacaNY, United States; ^19^Department of Plant Science, The Pennsylvania State University, University ParkPA, United States; ^20^Center for Organismal Studies, Heidelberg UniversityHeidelberg, Germany; ^21^Department of Plant Biology, University of California, Davis, DavisCA, United States; ^22^Department of Molecular and Cell Biology, University of California, Berkeley, BerkeleyCA, United States; ^23^Program in Bioinformatics and Computational Biology, The University of North Carolina, Chapel HillNC, United States; ^24^Plant Sciences (IBG-2), Forschungszentrum Jülich GmbH, JülichGermany; ^25^CIRAD, UMR AGAP, INRIA, VirtualPlantsMontpellier, France; ^26^National Institute for Mathematical and Biological Synthesis, University of Tennessee, Knoxville, KnoxvilleTN, United States; ^27^Department of Remote Sensing and Photogrammetry, Finnish Geospatial Research Institute, National Land Survey of FinlandMasala, Finland; ^28^Centre of Excellence in Laser Scanning Research, National Land Survey of FinlandMasala, Finland; ^29^Department of Ecology and Evolutionary Biology, University of Tennessee, Knoxville, KnoxvilleTN, United States; ^30^Unidad de Genómica Avanzada, Laboratorio Nacional de Genómica para la Biodiversidad, Center for Research and Advanced Studies of the National Polytechnic Institute (CINVESTAV)Irapuato, Mexico; ^31^Department of Botany, University of Wisconsin–Madison, MadisonWI, United States; ^32^Department of Plant and Soil Sciences and Delaware Biotechnology Institute, University of Delaware, NewarkDE, United States

**Keywords:** plant biology, plant science, morphology, mathematics, topology, modeling

## Abstract

The geometries and topologies of leaves, flowers, roots, shoots, and their arrangements have fascinated plant biologists and mathematicians alike. As such, plant morphology is inherently mathematical in that it describes plant form and architecture with geometrical and topological techniques. Gaining an understanding of how to modify plant morphology, through molecular biology and breeding, aided by a mathematical perspective, is critical to improving agriculture, and the monitoring of ecosystems is vital to modeling a future with fewer natural resources. In this white paper, we begin with an overview in quantifying the form of plants and mathematical models of patterning in plants. We then explore the fundamental challenges that remain unanswered concerning plant morphology, from the barriers preventing the prediction of phenotype from genotype to modeling the movement of leaves in air streams. We end with a discussion concerning the education of plant morphology synthesizing biological and mathematical approaches and ways to facilitate research advances through outreach, cross-disciplinary training, and open science. Unleashing the potential of geometric and topological approaches in the plant sciences promises to transform our understanding of both plants and mathematics.

## Introduction

### Morphology from the Perspective of Plant Biology

**FIGURE 1:**
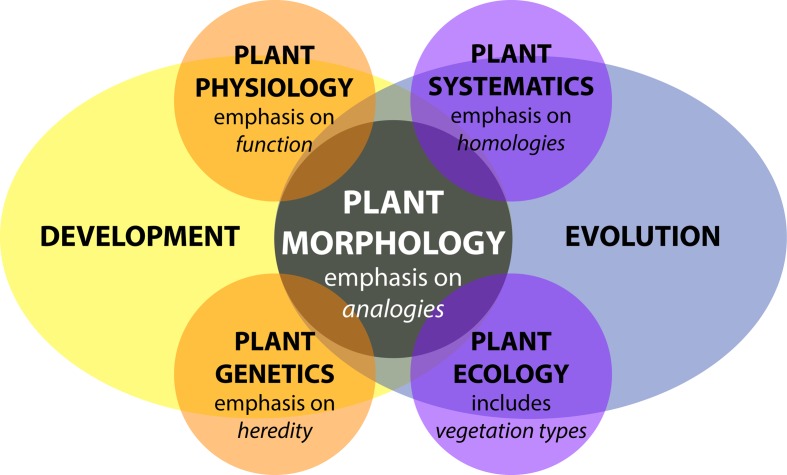
Plant morphology from the perspective of biology. Adapted from Kaplan (2001). Plant morphology interfaces with all disciplines of plant biology—plant physiology, plant genetics, plant systematics, and plant ecology—influenced by both developmental and evolutionary forces.

The study of plant morphology interfaces with all biological disciplines (**Figure 1**). Plant morphology can be descriptive and categorical, as in systematics, which focuses on biological homologies to discern groups of organisms (Mayr, 1981; Wiens, 2000). In plant ecology, the morphology of communities defines vegetation types and biomes, including their relationship to the environment. In turn, plant morphologies are mutually informed by other fields of study, such as plant physiology, the study of the functions of plants, plant genetics, the description of inheritance, and molecular biology, the underlying gene regulation (Kaplan, 2001).

Plant morphology is more than an attribute affecting plant organization, it is also dynamic. Developmentally, morphology reveals itself over the lifetime of a plant through varying rates of cell division, cell expansion, and anisotropic growth (Esau, 1960; Steeves and Sussex, 1989; Niklas, 1994). Response to changes in environmental conditions further modulate the abovementioned parameters. Development is genetically programmed and driven by biochemical processes that are responsible for physical forces that change the observed patterning and growth of organs (Green, 1999; Peaucelle et al., 2011; Braybrook and Jönsson, 2016). In addition, external physical forces affect plant development, such as heterogeneous soil densities altering root growth or flows of air, water, or gravity modulating the bending of branches and leaves (Moulia and Fournier, 2009). Inherited modifications of development over generations results in the evolution of plant morphology (Niklas, 1997). Development and evolution set the constraints for how the morphology of a plant arises, regardless of whether in a systematic, ecological, physiological, or genetic context (**Figure 1**).

### Plant Morphology from the Perspective of Mathematics

In 1790, Johann Wolfgang von Goethe pioneered a perspective that transformed the way mathematicians think about plant morphology: the idea that the essence of plant morphology is an underlying repetitive process of transformation (Goethe, 1790; Friedman and Diggle, 2011). The modern challenge that Goethe’s paradigm presents is to quantitatively describe transformations resulting from differences in the underlying genetic, developmental, and environmental cues. From a mathematical perspective, the challenge is how to define shape descriptors to compare plant morphology with topological and geometrical techniques and how to integrate these shape descriptors into simulations of plant development.

#### Mathematics to Describe Plant Shape and Morphology

**FIGURE 2:**
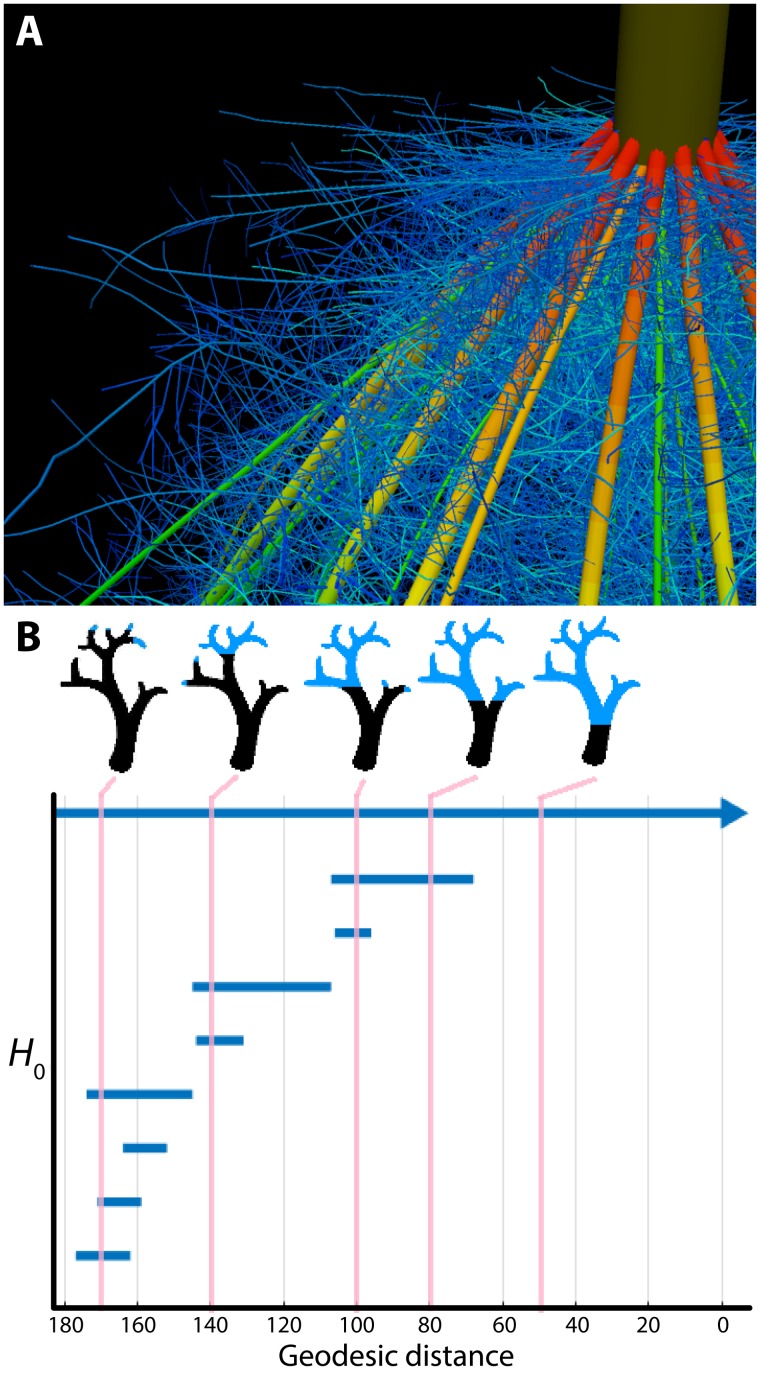
Plant morphology from the perspective of mathematics. **(A)** The topological complexity of plants requires a mathematical framework to describe and simulate plant morphology. Shown is the top of a maize crown root 42 days after planting. Color represents root diameter, revealing topology and different orders of root architecture. Image by Jennifer T. Yang and provided by JPL (Pennsylvania State University). **(B)** Persistent homology deforms a given plant morphology using functions to define self-similarity in a structure. In this example, a geodesic distance function is traversed to the ground level of a tree (that is, the shortest curved distance of each voxel to the base of the tree), as visualized in blue in successive images. The branching structure, as defined across scales of the geodesic distance function is recorded as an *H*_0_ (zero-order homology) barcode, which in persistent homology refers to connected components. As the branching structure is traversed by the function, connected components are “born” and “die” as terminal branches emerge and fuse together. Each of these components is indicated as a bar in the *H*_0_ barcode, and the correspondence of the barcode to different points in the function is indicated by vertical lines, in pink. Images provided by ML (Danforth Plant Science Center).

Several areas of mathematics can be used to extract quantitative measures of plant shape and morphology. One intuitive representation of the plant form relies on the use of skeletal descriptors that reduce the branching morphology of plants to a set of intersecting lines or curve segments, constituting a mathematical graph. These skeleton-based mathematical graphs can be derived from manual measurement (Godin et al., 1999; Watanabe et al., 2005) or imaging data (Bucksch et al., 2010; Aiteanu and Klein, 2014). Such skeletal descriptions can be used to derive quantitative measurements of lengths, diameters, and angles in tree crowns (Bucksch and Fleck, 2011; Raumonen et al., 2013; Seidel et al., 2015) and roots, at a single time point (Fitter, 1987; Danjon et al., 1999; Lobet et al., 2011; Galkovskyi et al., 2012) or over time to capture growth dynamics (Symonova et al., 2015). Having a skeletal description in place allows the definition of orders, in a biological and mathematical sense, to enable morphological analysis from a topological perspective (**Figure 2A**). Topological analyses can be used to compare shape characteristics independently of events that transform plant shape geometrically, providing a framework by which plant morphology can be modeled. The relationships between orders, such as degree of self-similarity (Prusinkiewicz, 2004) or self-nestedness (Godin and Ferraro, 2010) are used to quantitatively summarize patterns of plant morphology. Persistent homology (**Figure 2B**), an extension of Morse theory (Milnor, 1963), transforms a given plant shape gradually to define self-similarity (MacPherson and Schweinhart, 2012) and morphological properties (Edelsbrunner and Harer, 2010; Li et al., 2017) on the basis of topological event statistics. In the example in **Figure 2B**, topological events are represented by the geodesic distance at which branches are “born” and “die” along the length of the structure.

In the 1980s, David Kendall defined an elegant statistical framework to compare shapes (Kendall, 1984). His idea was to compare the outline of shapes in a transformation-invariant fashion. This concept infused rapidly as morphometrics into biology (Bookstein, 1997) and is increasingly carried out using machine vision techniques (Wilf et al., 2016). Kendall’s idea inspired the development of methods such as elliptical Fourier descriptors (Kuhl and Giardina, 1982) and new trends employing the Laplace Beltrami operator (Reuter et al., 2009), both relying on the spectral decompositions of shapes (Chitwood et al., 2012; Laga et al., 2014; Rellán-Álvarez et al., 2015). Beyond the organ level, such morphometric descriptors were used to analyze cellular expansion rates of rapidly deforming primordia into mature organ morphologies (Rolland-Lagan et al., 2003; Remmler and Rolland-Lagan, 2012; Das Gupta and Nath, 2015).

From a geometric perspective, developmental processes construct surfaces in a three-dimensional space. Yet, the embedding of developing plant morphologies into a three-dimensional space imposes constraints on plant forms. Awareness of such constraints has led to new interpretations of plant morphology (Prusinkiewicz and de Reuille, 2010; Bucksch et al., 2014b) that might provide avenues to explain symmetry and asymmetry in plant organs (e.g., Martinez et al., 2016) or the occurrence of plasticity as a morphological response to environmental changes (e.g., Royer et al., 2009; Palacio-López et al., 2015; Chitwood et al., 2016).

#### Mathematics to Simulate Plant Morphology

Computer simulations use principles from graph theory, such as graph rewriting, to model plant morphology over developmental time by successively augmenting a graph with vertices and edges as plant development unfolds. These rules unravel the differences between observed plant morphologies across plant species (Kurth, 1994; Prusinkiewicz et al., 2001; Barthélémy and Caraglio, 2007) and are capable of modeling fractal descriptions that reflect the repetitive and modular appearance of branching structures (Horn, 1971; Hallé, 1971, 1986). Recent developments in functional-structural modeling abstract the genetic mechanisms driving the developmental program of tree crown morphology into a computational framework (Runions et al., 2007; Palubicki et al., 2009; Palubicki, 2013). Similarly, functional-structural modeling techniques are utilized in root biology to simulate the efficiency of nutrient and water uptake following developmental programs (Nielsen et al., 1994; Dunbabin et al., 2013).

Alan Turing, a pioneering figure in 20th-century science, had a longstanding interest in phyllotactic patterns. Turing’s approach to the problem was twofold: first, a detailed geometrical analysis of the patterns (Turing, 1992), and second, an application of his theory of morphogenesis through local activation and long-range inhibition (Turing, 1952), which defined the first reaction-diffusion system for morphological modeling. Combining physical experiments with computer simulations, Douady and Coudert (1996) subsequently modeled a diffusible chemical signal produced by a developing primordium that would inhibit the initiation of nearby primordia, successfully recapitulating known phyllotactic patterns in the shoot apical meristem (Bernasconi, 1994; Meinhardt, 2004; Jönsson et al., 2005; Nikolaev et al., 2007; Hohm et al., 2010; Fujita et al., 2011), the number of floral organs (Kitazawa and Fujimoto, 2015), the regular spacing of root hairs (Meinhardt and Gierer, 1974), and the establishment of specific vascular patterns (Meinhardt, 1976).

## Emerging Questions and Barriers in the Mathematical Analysis of Plant Morphology

A true synthesis of plant morphology, which comprehensively models observed biological phenomena and incorporates a mathematical perspective, remains elusive. In this section, we highlight current focuses in the study of plant morphology, including the technical limits of acquiring morphological data, phenotype prediction, responses of plants to the environment, models across biological scales, and the integration of complex phenomena, such as fluid dynamics, into plant morphological models.

### Technological Limits to Acquiring Plant Morphological Data

**FIGURE 3:**
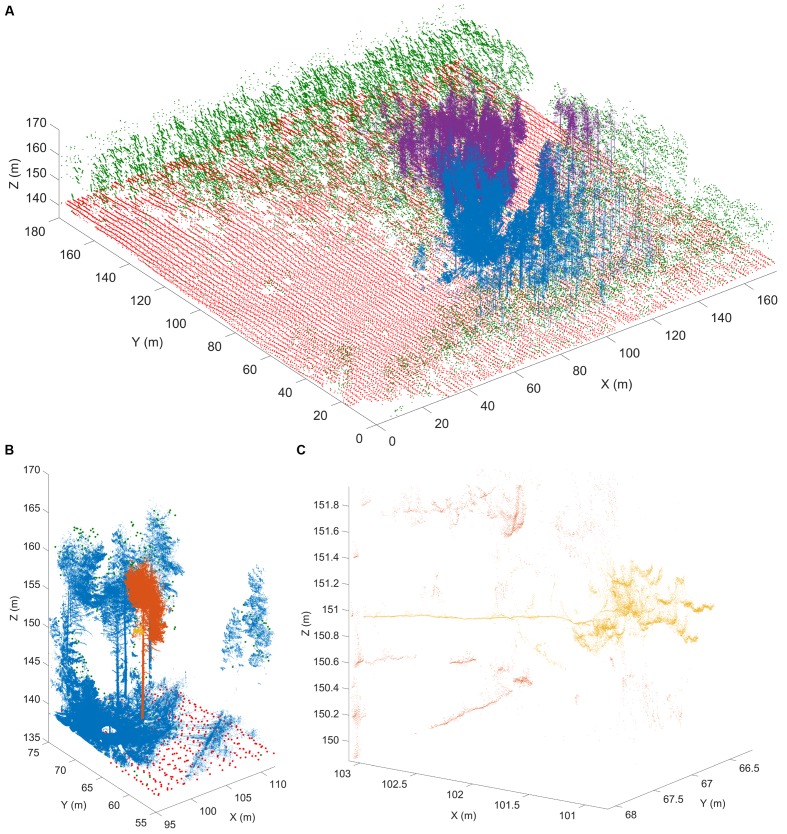
Terrestrial laser scanning creates a point cloud reconstruction of a Finnish forest. **(A)** Structure of a boreal forest site in Finland as seen with airborne (ALS) and terrestrial (TLS) laser scanning point clouds. The red (ground) and green (above-ground) points are obtained from National Land Survey of Finland national ALS point clouds that cover hundreds of thousands of square kilometers with about 1 point per square meter resolution. The blue and magenta point clouds are results of two individual TLS measurements and have over 20 million points each within an area of about 500 m^2^. TLS point density varies with range but can be thousands of points per square meter up to tens of meters away from the scanner position. **(B)** An excerpt from a single TLS point cloud (blue). The TLS point cloud is so dense that individual tree point clouds (orange) and parts from them (yellow) can be selected for detailed analysis. **(C)** A detail from a single TLS point cloud. Individual branches (yellow) 20 m above ground can be inspected from the point cloud with centimeter level resolution to estimate their length and thickness. Images provided by EP (Finnish Geospatial Research Institute in the National Land Survey of Finland). ALS data was obtained from the National Land Survey of Finland Topographic Database, 08/2012 (National Land Survey of Finland open data license, version 1.0).

There are several technological limits to acquiring plant morphological data that must be overcome to move this field forward. One such limitation is the acquisition of quantitative plant images. Many acquisition systems do not provide morphological data with measurable units. Approaches that rely on the reflection of waves from the plant surface can provide quantitative measurements for morphological analyses. Time of flight scanners, such as terrestrial laser scanning, overcome unit-less measurement systems by recording the round-trip time of hundreds of thousands of laser beams sent at different angles from the scanner to the first plant surface within the line of sight (Vosselman and Maas, 2010) (**Figure 3**). Leveraging the speed of light allows calculation of the distance between a point on the plant surface and the laser scanner.

Laser scanning and the complementary, yet unitless, approach of stereovision both produce surface samples or point clouds as output. However, both approaches face algorithmic challenges encountered when plant parts occlude each other, since both rely on the reflection of waves from the plant surface (Bucksch, 2014). Radar provides another non-invasive technique to study individual tree and forest structures over wide areas. Radar pulses can either penetrate or reflect from foliage, depending on the selected wavelength (Kaasalainen et al., 2015). Most radar applications occur in forestry and are being operated from satellites or airplanes. Although more compact and agile systems are being developed for precision forestry above- and below-ground (Feng et al., 2016), their resolution is too low to acquire the detail in morphology needed to apply hierarchy or similarity oriented mathematical analysis strategies.

**FIGURE 4:**
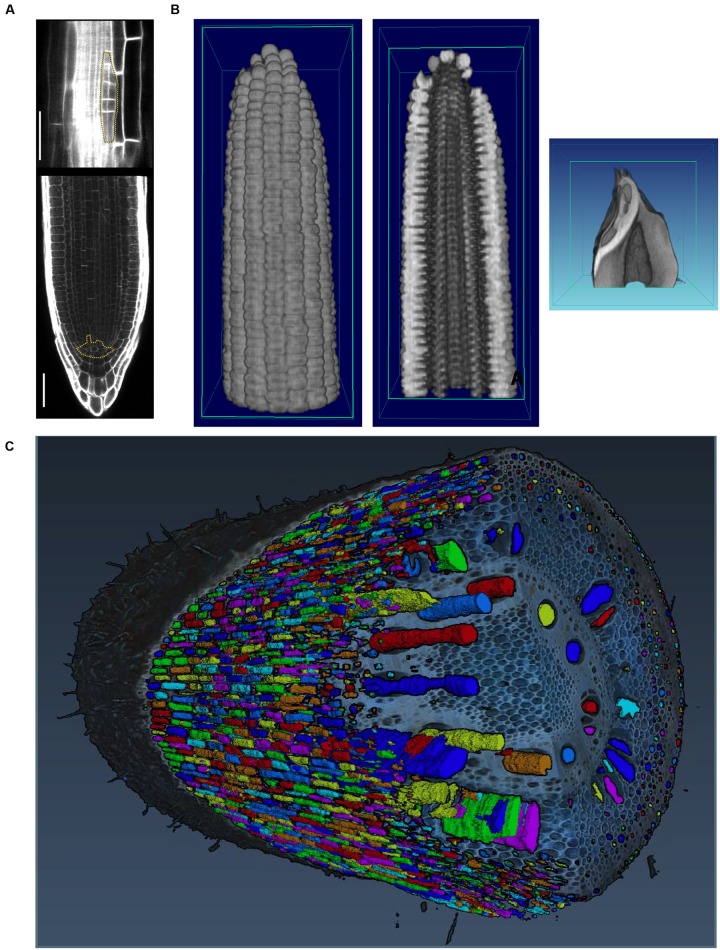
Imaging techniques to capture plant morphology. **(A)** Confocal sections of an Arabidopsis root. The upper panel shows a new lateral root primordium at an early stage of development (highlighted in yellow). At regular intervals new roots branch from the primary root. The lower panel shows the primary root meristem and the stem cell niche (highlighted in yellow) from which all cells derive. Scale bars: 100 μm. Images provided by AM (Heidelberg University). **(B)** Computational tomographic (CT) x-ray sections through a reconstructed maize ear (left and middle) and kernel (right). Images provided by CT (Donald Danforth Plant Science Center). **(C)** Laser ablation tomography (LAT) image of a nodal root from a mature, field-grown maize plant, with color segmentation showing definition of cortical cells, aerenchyma lacunae, and metaxylem vessels. Image by Jennifer T. Yang and provided by JPL (Pennsylvania State University).

Image acquisition that resolves occlusions by penetrating plant tissue is possible with X-ray (Kumi et al., 2015) and magnetic resonance imaging (MRI; van Dusschoten et al., 2016). While both technologies resolve occlusions and can even penetrate soil, their limitation is the requirement of a closed imaging volume. Thus, although useful for a wide array of purposes, MRI and X-ray are potentially destructive if applied to mature plant organs such as roots in the field or tree crowns that are larger than the imaging volume (Fiorani et al., 2012). Interior plant anatomy can be imaged destructively using confocal microscopy and laser ablation (**Figure 4**) or nano- or micro-CT tomography techniques, that are limited to small pot volumes, to investigate the first days of plant growth.

### The Genetic Basis of Plant Morphology

One of the outstanding challenges in plant biology is to link the inheritance and activity of genes with observed phenotypes. This is particularly challenging for the study of plant morphology, as both the genetic landscape and morphospaces are complex: modeling each of these phenomena alone is difficult, let alone trying to model morphology as a result of genetic phenomena (Benfey and Mitchell-Olds, 2008; Lynch and Brown, 2012; Chitwood and Topp, 2015). Although classic examples exist in which plant morphology is radically altered by the effects of a few genes (Doebley, 2004; Clark et al., 2006; Kimura et al., 2008), many morphological traits have a polygenic basis (Langlade et al., 2005; Tian et al., 2011; Chitwood et al., 2013).

Quantitative trait locus (QTL) analyses can identify the polygenic basis for morphological traits that span scales from the cellular to the whole organ level. At the cellular level, root cortex cell number (Ron et al., 2013), the cellular basis of carpel size (Frary et al., 2000), and epidermal cell area and number (Tisné et al., 2008) have been analyzed. The genetic basis of cellular morphology ultimately affects organ morphology, and quantitative genetic bases for fruit shape (Paran and van der Knaap, 2007; Monforte et al., 2014), root morphology (Zhu et al., 2005; Clark et al., 2011; Topp et al., 2013; Zurek et al., 2015), shoot apical meristem shape (Leiboff et al., 2015; Thompson et al., 2015), leaf shape (Langlade et al., 2005; Ku et al., 2010; Tian et al., 2011; Chitwood et al., 2014a,b; Zhang et al., 2014; Truong et al., 2015), and tree branching (Kenis and Keulemans, 2007; Segura et al., 2009) have been described.

Natural variation in cell, tissue, or organ morphology ultimately impacts plant physiology, and vice versa. For example, formation of root cortical aerenchyma was linked to better plant growth under conditions of suboptimal availability of water and nutrients (Zhu et al., 2010; Postma and Lynch, 2011; Lynch, 2013), possibly because aerenchyma reduces the metabolic costs of soil exploration. Maize genotypes with greater root cortical cell size or reduced root cortical cell file number reach greater depths to increase water capture under drought conditions, possibly because those cellular traits reduce metabolic costs of root growth and maintenance (Chimungu et al., 2015). The control of root angle that results in greater water capture in rice as water tables recede was linked to the control of auxin distribution (Uga et al., 2013). Similarly, in shoots, natural variation can be exploited to find genetic loci that control shoot morphology, e.g., leaf erectness (Ku et al., 2010; Feng et al., 2011).

High-throughput phenotyping techniques are increasingly used to reveal the genetic basis of natural variation (Tester and Langridge, 2010). In doing so, phenotyping techniques complement classic approaches of reverse genetics and often lead to novel insights, even in a well-studied species like *Arabidopsis thaliana*. Phenotyping techniques have revealed a genetic basis for dynamic processes such as root growth (Slovak et al., 2014) and traits that determine plant height (Yang et al., 2014). Similarly, high-resolution sampling of root gravitropism has led to an unprecedented understanding of the dynamics of the genetic basis of plasticity (Miller et al., 2007; Brooks et al., 2010; Spalding and Miller, 2013).

### The Environmental Basis of Plant Morphology

Phenotypic plasticity is defined as the ability of one genotype to produce different phenotypes based on environmental differences (Bradshaw, 1965; DeWitt and Scheiner, 2004) and adds to the phenotypic complexity created by genetics and development. Trait variation in response to the environment has been analyzed classically using ‘reaction norms,’ where the phenotypic value of a certain trait is plotted for two different environments (Woltereck, 1909). If the trait is not plastic, the slope of the line connecting the points will be zero; if the reaction norm varies across the environment the trait is plastic and the slope of the reaction norm line will be a measure of the plasticity. As most of the responses of plants to their environment are non-linear, more insight into phenotypic plasticity can be obtained by analyzing dose-response curves or dose-response surfaces (Mitscherlich, 1909; Poorter et al., 2010).

Seminal work by Clausen et al. (1941) demonstrated using several clonal species in a series of reciprocal transplants that, although heredity exerts the most measureable effects on plant morphology, environment is also a major source of phenotypic variability. Research continues to explore the range of phenotypic variation expressed by a given genotype in the context of different environments, which has important implications for many fields, including conservation, evolution, and agriculture (Nicotra et al., 2010; DeWitt, 2016). Many studies examine phenotypes across latitudinal or altitudinal gradients, or other environmental clines, to characterize the range of possible variation and its relationship to the process of local adaptation (Cordell et al., 1998; Díaz et al., 2016).

**FIGURE 5:**
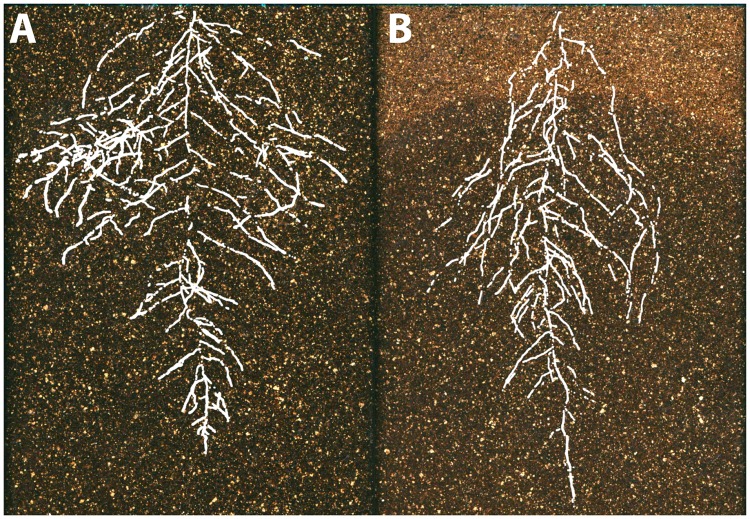
The environmental basis of plant morphology. Root system architecture of *Arabidopsis* Col-0 plants expressing ProUBQ10:LUC2o growing in **(A)** control and **(B)** water-deficient conditions using the GLO-Roots system (Rellán-Álvarez et al., 2015). Images provided by RR-Á (Laboratorio Nacional de Genómica para la Biodiversidad, CINVESTAV) are a composite of a video originally published (Rellán-Álvarez et al., 2015).

Below-ground, plants encounter diverse sources of environmental variability, including water availability, soil chemistry, and physical properties like soil hardness and movement. These factors vary between individual plants (Razak et al., 2013) and within an individual root system, where plants respond at spatio-temporal levels to very different granularity (Drew, 1975; Robbins and Dinneny, 2015). Plasticity at a micro-environmental scale has been linked to developmental and molecular mechanisms (Bao et al., 2014). The scientific challenge here is to integrate these effects at a whole root system level and use different scales of information to understand the optimal acquisition in resource limited conditions (Rellán-Álvarez et al., 2016) (**Figure 5**).

### Integrating Models from Different Levels of Organization

Since it is extremely difficult to examine complex interdependent processes occurring at multiple spatio-temporal scales, mathematical modeling can be used as a complementary tool with which to disentangle component processes and investigate how their coupling may lead to emergent patterns at a systems level (Hamant et al., 2008; Band and King, 2012; Band et al., 2012; Jensen and Fozard, 2015). To be practical, a multiscale model should generate well-constrained predictions despite significant parameter uncertainty (Gutenkunst et al., 2007; Hofhuis et al., 2016). It is desirable that a multiscale model has certain modularity in its design such that individual modules are responsible for modeling specific spatial aspects of the system (Baldazzi et al., 2012). Imaging techniques can validate multiscale models (e.g., Willis et al., 2016) such that simulations can reliably guide experimental studies.

**FIGURE 6:**
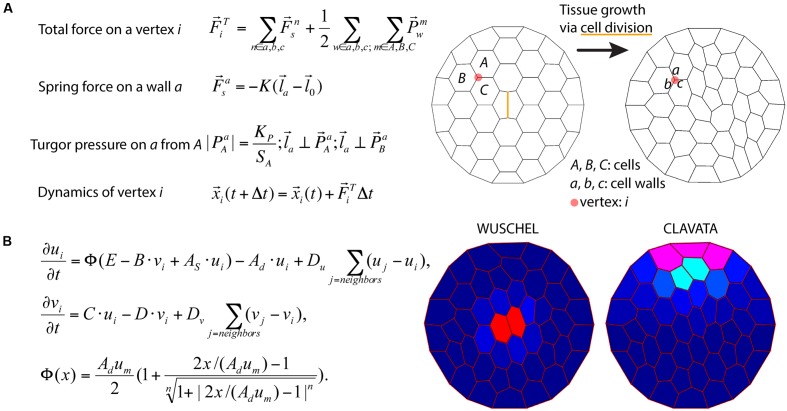
Integration of tissue growth and reaction-diffusion models. **(A)** Vertex model of cellular layers (Prusinkiewicz and Lindenmayer, 1990). *K*, 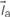, and 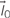 are the spring constant, current length, and rest length for wall *a. K*_P_ is a constant and *S*_A_ is the size of cell *A*. Δt is time step. Shown is a simulation of cell network growth. **(B)** Reaction diffusion model of the shoot apical meristem for WUSCHEL and CLAVATA interactions (Fujita et al., 2011). *u* = WUS, *v* = CLV, *i* = cell index, Φ is a sigmoid function. *E*, *B*, *A*_S_, *A*_d_, *C*, *D*, *u*_m_, *D*_u_, *D*_v_ are positive constants. Shown are the distributions of WUS and CLV levels within a dynamic cell network. Images provided by DB (Virginia Tech).

To illustrate the challenges of multi-scale modeling, we highlight an example that encompasses molecular and cellular scales. At the molecular scale, models can treat some biomolecules as diffusive, but others, such as membrane-bound receptors, can be spatially restricted (Battogtokh and Tyson, 2016). Separately, at the cellular scale, mathematical models describe dynamics of cell networks where the mechanical pressures exerted on the cell walls are important factors for cell growth and division (Jensen and Fozard, 2015) (**Figure 6A**). In models describing plant development in a two-dimensional cross-section geometry, cells are often modeled as polygons defined by walls between neighboring cells. The spatial position of a vertex, where the cell walls of three neighboring cells coalesce, is a convenient variable for mathematical modeling of the dynamics of cellular networks (Prusinkiewicz and Runions, 2012). A multiscale model can then be assembled by combining the molecular and cellular models. Mutations and deletions of the genes encoding the biomolecules can be modeled by changing parameters. By inspecting the effects of such modifications on the dynamics of the cellular networks, the relationship between genotypes and phenotypes can be predicted. For example, Fujita et al. (2011) model integrates the dynamics of cell growth and division with the spatio-temporal dynamics of the proteins involved in stem cell regulation and simulates shoot apical meristem development in wild type and mutant plants (**Figure 6B**).

### Modeling the Impact of Morphology on Plant Function

**FIGURE 7:**
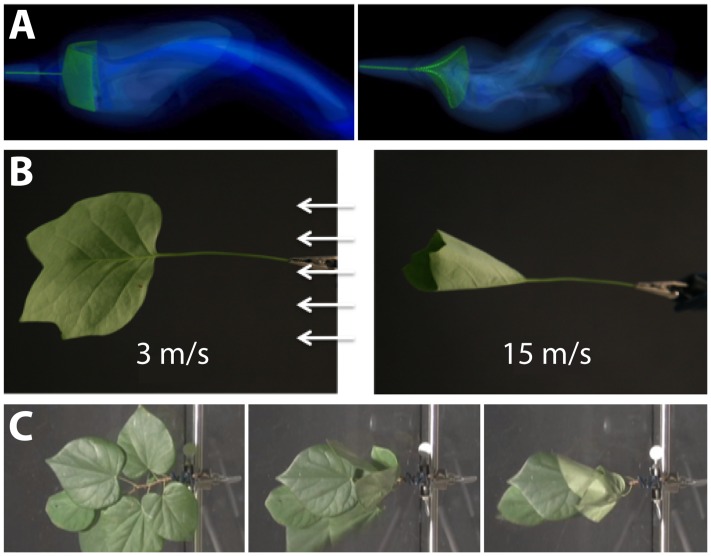
Modeling the interaction between plant morphology and fluid dynamics. **(A)** 3D immersed boundary simulations of flow past a flexible rectangular sheet (left) and disk with a cut from the center to edge (right). Both structures are attached to a flexible petiole, and the flow is from left to right. The contours show the magnitude of vorticity (the rotation in the air). The circular disk reconfigures into a cone shape, similar to many broad leaves. **(B)** Reconfiguration of tulip poplar leaves in 3 m/s (left) and 15 m/s flow (right). The leaves typically flutter at lower wind speeds and reconfigure into stable cones at high wind speeds. **(C)** A cluster of redbud leaves in wind moving from right to left. The wind speed is increased from 3 m/s (left) to 6 m/s (middle) and 12 m/s (right). Note that the entire cluster reconfigures into a cone shape. This is different from the case of tulip poplars and maples where each leaf individually reconfigures into a conic shape. Images provided by LM (University of North Carolina, Chapel Hill, NC, United States).

Quantitative measures of plant morphology are critical to understand function. Vogel (1989) was the first to provide quantitative data that showed how shape changes in leaves reduce drag or friction in air or water flows. He found that single broad leaves reconfigure at high flow velocities into cone shapes to reduce flutter and drag (**Figures 7A,B**). More recent work discovered that the cone shape is significantly more stable than other reconfigurations such as U-shapes (Miller et al., 2012). Subsequent experimental studies on broad leaves, compound leaves, and flowers also support rapid repositioning in response to strong currents as a general mechanism to reduce drag (Niklas, 1992; Ennos, 1997; Etnier and Vogel, 2000; Vogel, 2006) (**Figure 7C**). It is a combination of morphology and anatomy, and the resultant material properties, which lead to these optimal geometric re-configurations of shape.

From a functional perspective, it is highly plausible that leaf shape and surface-material properties alter the boundary layer of a fluid/gas over the leaf surface or enhance passive movement that can potentially augment gas and heat exchange. For example, it has been proposed that the broad leaves of some trees flutter for the purpose of convective and evaporative heat transfer (Thom, 1968; Grant, 1983). Any movement of the leaf relative to the movement of the air or water may decrease the boundary layer and increase gas exchange, evaporation, and heat dissipation (Roden and Pearcy, 1993). Each of these parameters may be altered by the plant to improve the overall function of the leaf (Vogel, 2012).

The growth of the plant continuously modifies plant topology and geometry, which in turn changes the balance between organ demand and production. At the organismal scale, the 3D spatial distribution of plant organs is the main interface between the plant and its environment. For example, the 3D arrangement of branches impacts light interception and provides the support for different forms of fluxes (water, sugars) and signals (mechanical constraints, hormones) that control plant functioning and growth (Godin and Sinoquet, 2005).

## Milestones in Education and Outreach to Accelerate the Infusion of Math into the Plant Sciences

Mathematics and plant biology need to interact more closely to accelerate scientific progress. Opportunities to interact possibly involve cross-disciplinary training, workshops, meetings, and funding opportunities. In this section, we outline perspectives for enhancing the crossover between mathematics and plant biology.

### Education

Mathematics has been likened to “biology’s next microscope,” because of the insights into an otherwise invisible world it has to offer. Conversely, biology has been described as “mathematics’ next physics,” stimulating novel mathematical approaches because of the hitherto unrealized phenomena that biology studies (Cohen, 2004). The scale of the needed interplay between mathematics and plant biology is enormous and may lead to new science disciplines at the interface of both: ranging from the cellular, tissue, organismal, and community levels to the global; touching upon genetic, transcriptional, proteomic, metabolite, and morphological data; studying the dynamic interactions of plants with the environment or the evolution of new forms over geologic time; and spanning quantification, statistics, and mechanistic mathematical models.

Research is becoming increasingly interdisciplinary, and undergraduate, graduate, and post-graduate groups are actively trying to bridge the gap between mathematics and biology skillsets. While many graduate programs have specialization tracks under the umbrella of mathematics or biology-specific programs, increasingly departments are forming specially designed graduate groups for mathematical/quantitative biology^1^^,^^2^ to strengthen the interface between both disciplines. This will necessitate team-teaching across disciplines to train the next generation of mathematical/computational plant scientists.

### Public Outreach: Citizen Science and the Maker Movement

Citizen science, which is a method to make the general public aware of scientific problems and employ their help in solving them^3^, is an ideal platform to initiate a synthesis between plant biology and mathematics because of the relatively low cost and accessibility of each field. Arguably, using citizen science to collect plant morphological diversity has already been achieved, but has yet to be fully realized. In total, it is estimated that the herbaria of the world possess greater than 207 million voucher specimens^4^, representing the diverse lineages of land plants collected over their respective biogeographies over a timespan of centuries. Digital documentation of the millions of vouchers held by the world’s botanic gardens is actively underway, allowing for researchers and citizens alike to access and study for themselves the wealth of plant diversity across the globe and centuries (Smith et al., 2003; Corney et al., 2012; Ryan, 2013).

The developmental changes in plants responding to environmental variability and microclimatic changes over the course of a growing season can be analyzed by studying phenology. Citizen science projects such as the USA National Phenology Network^5^ or Earthwatch^6^ and associated programs such as My Tree Tracker^7^ document populations and individual plants over seasons and years, providing a distributed, decentralized network of scientific measurements to study the effects of climate change on plants.

Citizen science is also enabled by low-cost, specialized equipment. Whether programming a camera to automatically take pictures at specific times or automating a watering schedule for a garden, the maker movement—a do-it-yourself cultural phenomenon that intersects with hacker culture—focuses on building custom, programmable hardware, whether via electronics, robotics, 3D-printing, or time-honored skills such as metal- and woodworking. The focus on programming is especially relevant for integrating mathematical approaches with plant science experiments. The low-cost of single-board computers like Raspberry Pi, HummingBoard, or CubieBoard is a promising example of how to engage citizen scientists into the scientific process and enable technology solutions to specific questions.

### Workshops and Funding Opportunities

**FIGURE 8:**
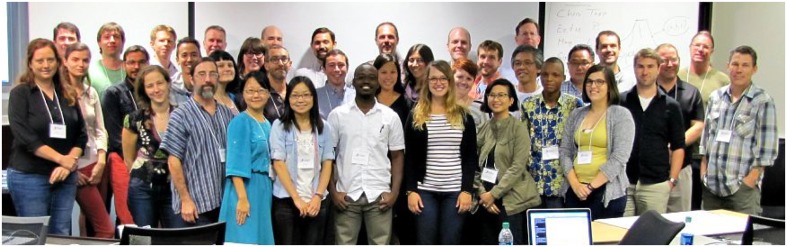
Milestones to accelerate the infusion of math into the plant sciences. Group photo of the authors from the National Institute for Mathematical and Biological Synthesis (NIMBioS) meeting on plant morphological models (University of Tennessee, Knoxville, September 2–4, 2015) that inspired this manuscript. Workshops such as these, bringing mathematicians and plant biologists together, will be necessary to create a new synthesis of plant morphology.

Simply bringing mathematicians and plant biologists together to interact, to learn about new tools, approaches, and opportunities in each discipline is a major opportunity for further integration of these two disciplines and strengthen new disciplines at the interface of both. This white paper itself is a testament to the power of bringing mathematicians and biologists together, resulting from a National Institute for Mathematical and Biological Synthesis (NIMBioS) workshop titled “Morphological Plant Modeling: Unleashing Geometric and Topologic Potential within the Plant Sciences,” held at the University of Tennessee, Knoxville, September 2–4, 2015^8^ (**Figure 8**). Other mathematical institutes such as the Mathematical Biology Institute (MBI) at Ohio State University^9^, the Statistical and Applied Mathematical Sciences Institute (SAMSI) in Research Triangle Park^10^, the Institute for Mathematics and Its Applications at University of Minnesota^11^, and the Centre for Plant Integrative Biology at the University of Nottingham^12^ have also hosted workshops for mathematical and quantitative biologists from the undergraduate student to the faculty level.

There are efforts to unite biologists and mathematics through initiatives brought forth from The National Science Foundation, including Mathematical Biology Programs^13^ and the Joint DMS/NIGMS Initiative to Support Research at the Interface of the Biological and Mathematical Sciences^14^ (DMS/NIGMS). Outside of the Mathematics and Life Sciences Divisions, the Division of Physics houses a program on the Physics of Living Systems. Societies such as The Society for Mathematical Biology and the Society for Industrial and Applied Mathematics (SIAM) Life Science Activity Group^15^ are focused on the dissemination of research at the intersection of math and biology, creating many opportunities to present research and provide funding. We emphasize the importance that funding opportunities have had and will continue to have in the advancement of plant morphological modeling.

### Open Science

Ultimately, mathematicians, computational scientists, and plant biology must unite at the level of jointly collecting data, analyzing it, and doing science together. Open and timely data sharing to benchmark code is a first step to unite these disciplines along with building professional interfaces to bridge between the disciplines (Bucksch et al., 2017; Pradal et al., 2017).

A number of platforms provide open, public access to datasets, figures, and code that can be shared, including Dryad^16^, Dataverse^17^, and Figshare^18^. Beyond the ability to share data is the question of open data formats and accessibility. For example, in remote sensing research it is unfortunately common that proprietary data formats are used, which prevents their use without specific software. This severely limits the utility and community building aspects of plant morphological research. Beyond datasets, making code openly available, citable, and user-friendly is a means to share methods to analyze data. Places to easily share code include web-based version controlled platforms like Bitbucket^19^ or Github^20^ and software repositories like Sourceforge^21^. Furthermore, numerous academic Journals (e.g., Nature Methods, Applications in Plant Sciences, and Plant Methods) already accept publications that focus on methods and software to accelerate new scientific discovery (Pradal et al., 2013).

Meta-analysis datasets provide curated resources where numerous published and unpublished datasets related to a specific problem (or many problems) can be accessed by researchers^22^. The crucial element is that data is somehow reflective of universal plant morphological features, bridging the gap between programming languages and biology, as seen in the Root System Markup Language (Lobet et al., 2015) and OpenAlea (Pradal et al., 2008, 2015). Bisque is a versatile platform to store, organize, and analyze image data, providing simultaneously open access to data and analyses as well as the requisite computation (Kvilekval et al., 2010). CyVerse^23^ (formerly iPlant) is a similar platform, on which academic users get 100 GB storage for free and can create analysis pipelines that can be shared and reused (Goff et al., 2011). For example, DIRT^24^ is an automatic, high throughput computing platform (Bucksch et al., 2014a; Das et al., 2015) that the public can use hosted on CyVerse using the Texas Advanced Computing Center^25^ (TACC) resources at UT Austin that robustly extracts root traits from digital images. The reproducibility of these complex computational experiments can be improved using scientific workflows that capture and automate the exact methodology followed by scientists (Cohen-Boulakia et al., 2017). We emphasize here the importance of adopting open science policies at the individual investigator and journal level to continue strengthening the interface between plant and mathematically driven sciences.

## Conclusion: Unleashing Geometric and Topological Potential within the Plant Sciences

Plant morphology is a mystery from a molecular and quantification point of view. Hence, it fascinates both mathematical and plant biology researchers alike. As such, plant morphology holds the secret by which predetermined variations of organizational patterns emerge as a result of evolutionary, developmental, and environmental responses.

The persistent challenge at the intersection of plant biology and mathematical sciences might be the integration of measurements across different scales of the plant. We have to meet this challenge to derive and validate mathematical models that describe plants beyond the visual observable. Only then we will be able to modify plant morphology through molecular biology and breeding as means to develop needed agricultural outputs and sustainable environments for everybody.

Cross-disciplinary training of scientists, citizen science, and open science are inevitable first steps to develop the interface between mathematical-driven and plant biology-driven sciences. The result of these steps will be new disciplines, that will add to the spectrum of researchers in plant biology. Hence, to unleash the potential of geometric and topological approaches in the plant sciences, we need an interface familiar with both plants and mathematical approaches to meet the challenges posed by a future with uncertain natural resources as a consequence of climate change.

## Author Contributions

AB and DC conceived, wrote, and organized the manuscript. All authors contributed to writing the manuscript.

## Conflict of Interest Statement

The authors declare that the research was conducted in the absence of any commercial or financial relationships that could be construed as a potential conflict of interest.

## Footnotes

1 BioQuant at University of Heidelberg, http://www.bioquant.uni-heidelberg.de (retrieved February 28, 2017)

2 Quantitative Biosciences at Georgia Tech in Atlanta, http://qbios.gatech.edu (retrieved February 28, 2017)

3 For example, see the White Paper on Citizen Science for Europe, http://www.socientize.eu/sites/default/files/white-paper_0.pdf (retrieved May 29, 2016)

4 List of herbaria, https://en.wikipedia.org/wiki/List_of_herbaria (retrieved May 29, 2016)

5 https://www.usanpn.org/# (retrieved May 29, 2016)

6 http://earthwatch.org/scientific-research/special-initiatives/urban-resiliency (retrieved May 29, 2016)

7 http://www.mytreetracker.org/cwis438/websites/MyTreeTracker/About.php?WebSiteID=23 (retrieved May 29, 2016)

8 http://www.nimbios.org/workshops/WS_plantmorph (retrieved May 29, 2016)

9 https://mbi.osu.edu/ (retrieved May 29, 2016)

10 http://www.samsi.info/ (retrieved May 29, 2016)

11 https://www.ima.umn.edu/ (retrieved May 29, 2016)

12 https://www.cpib.ac.uk/outreach/cpib-summer-school/ (retrieved May 29, 2016)

13 https://www.nsf.gov/funding/pgm_summ.jsp?pims_id=5690 (retrieved May 29, 2016)

14 http://www.nsf.gov/funding/pgm_summ.jsp?pims_id=5300&org=DMS (retrieved May 29, 2016)

15 https://www.siam.org/activity/life-sciences/ (retrieved May 29, 2016)

16 http://datadryad.org/ (retrieved May 29, 2016)

17 http://dataverse.org/ (retrieved May 29, 2016)

18 https://figshare.com/ (retrieved May 29, 2016)

19 https://bitbucket.org/ (retrieved May 29, 2016)

20 https://github.com/ (retrieved May 29, 2016)

21 https://sourceforge.net/ (retrieved May 29, 2016)

22 BAAD: a Biomass And Allometry Database for woody plants, https://github.com/dfalster/baad (retrieved May 29, 2016)

23 http://www.cyverse.org/ (retrieved August 20, 2016)

24 http://dirt.iplantcollaborative.org/ (retrieved August 20, 2016)

25 https://www.tacc.utexas.edu/ (retrieved August 20, 2016)
